# Diabetes mellitus status modifies the association between N-terminal B-type natriuretic peptide and all-cause mortality risk in ischemic heart failure: a prospective cohort study

**DOI:** 10.1186/s13098-023-01046-5

**Published:** 2023-04-11

**Authors:** Weida Qiu, Anping Cai, Xiaoju Xiao, Zhiping Gao, Yingqing Feng, Liwen Li

**Affiliations:** 1 Department of Cardiology, Hypertension Research Laboratory, Guangdong Cardiovascular Institute, Guangdong Provincial People’s Hospital, Guangdong Academy of Medical Sciences, Southern Medical University, Guangzhou, China; 2 The Second School of Clinical Medicine, Southern Medical University, Guangzhou, China; 3 Concord medical center, Guangdong Provincial People’s Hospital, Guangdong Academy of Medical Sciences, Guangzhou, China; 4 Guangdong Provincial People’s Hospital, No. 106, Zhongshan 2nd Road, Yuexiu District, Guangzhou, 510080 China

**Keywords:** Diabetes mellitus, NT-proBNP, Ischemic heart failure, All-cause mortality

## Abstract

**Background:**

N-terminal B-type natriuretic peptide (NT-proBNP) discriminates mortality risk in diabetes mellitus (DM) and in heart failure (HF) populations. Whether DM status modifies the association between NT-proBNP and all-cause mortality risk in ischemic HF is unknown.

**Methods:**

This was a single-center, prospective cohort study conducted with 2287 ischemic HF patients. Subjects were divided into with DM group and without DM group. Multivariate Cox proportional-hazards models were conducted to calculate the hazard ratios (HRs) and 95% confidence intervals (CIs). The product of DM status and NT-proBNP were used to assess the interaction. Propensity score matching analysis was used to verify the robustness of the results.

**Results:**

Of 2287 ischemic HF participants, 1172 (51.2%) had DM. After a median follow-up of 3.19 years (7287 person-years), 479 (20.9%) of the participants died. After adjusting for the covariates, continuous NT-proBNP was more prominently associated with risk of mortality in HF patients with DM (HR: 1.65, 95% CI: 1.43–1.91) than those without (HR: 1.28, 95% CI: 1.09–1.50). A significant interaction of DM status and NT-proBNP was observed (P-interaction = 0.016). The relationships were consistent when NT-proBNP was considered as a categorical variable and in the propensity matching analysis.

**Conclusions:**

DM status modified the association between NT-proBNP and all-cause mortality in ischemic HF patients, suggesting that NT-proBNP was more prominently associated with risk of mortality in patients with DM than those without. Future studies to clarify the mechanisms underlying these observations are needed.

**Supplementary Information:**

The online version contains supplementary material available at 10.1186/s13098-023-01046-5.

## Introduction

Diabetes mellitus (DM) and heart failure (HF) commonly co-exist, and the presence of type 2 DM increases the incidence of HF and subsequent events [1–5]. The prevalence of DM and HF is projected to further increase with aging populations and lifestyle changes [6–8]. Improvement in prognosis prediction in HF patients complicated with DM is a top priority.

N-terminal B-type natriuretic peptide (NT-proBNP) is of greatest importance in the diagnosis and prognosis prediction in HF patients. Previous studies consistently demonstrated a clear relationship between NT-proBNP and subsequent risk of adverse cardiovascular outcomes [9, 10]. Recently, a report from a community-based cohort study consisting of 5861 subjects showed that NT-proBNP alone discriminated mortality risk better than traditional risk factors in people with DM. While in individuals without DM, the predicting ability of NT-proBNP was similar to the conventional risk factors, suggesting that NT-proBNP performed especially well in predicting death in general DM populations [11]. However, whether the association of NT-proBNP and all-cause mortality in HF patients varies by DM status remains unknown.

Accordingly, we hypothesized that DM status might modify the association between NT-proBNP and mortality risk in HF patients. Leveraging an ongoing prospective ischemic HF cohort, we aimed to evaluate the relationship between NT-proBNP and all-cause mortality among patients with and without DM.

## Methods

### Study design and participants

**Fig. 1:**
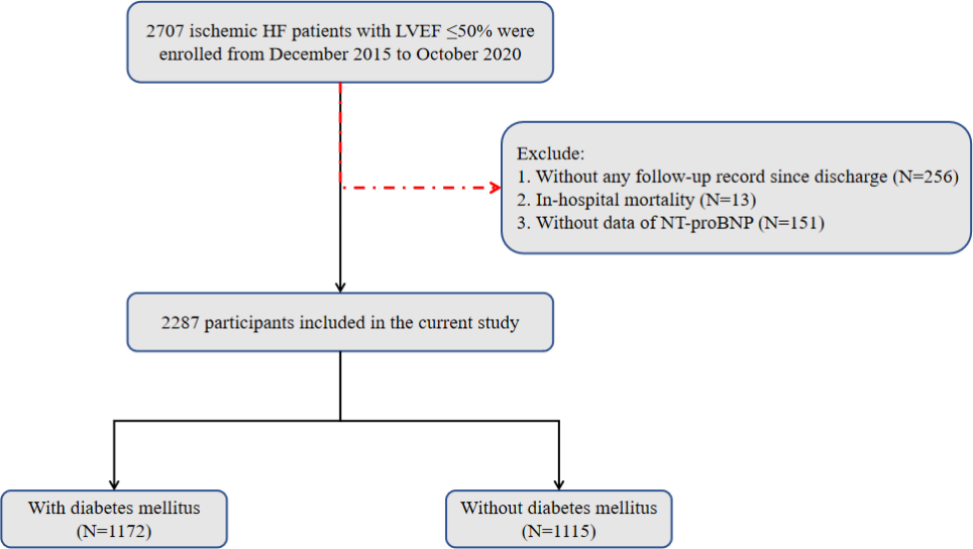
Study Flowchart. HF, heart failure; LVEF, left ventricular ejection fraction; NT-proBNP, N-terminal B-type natriuretic peptide

Designs and details of this prospective, single-center, ischemic HF cohort study have been described previously [12, 13]. Briefly, patients hospitalized at the department of cardiology of Guangdong Provincial People’s Hospital from December 2015 to October 2020 were screened and enrolled after informed consent was obtained. Ischemic HF was defined as prior myocardial infarction (MI) or prior revascularization, or being confirmed by coronary angiography during hospitalization, and left ventricular ejection fraction (LVEF) ≤ 50% evaluated by echocardiography (subjects with LVEF ≤ 45% were screened before 2019). Patients who were without NT-proBNP data, lost to follow-up, or died in the hospital were excluded (Fig. 1). This study was approved by the Clinical Research Ethics Committee of Guangdong Provincial People’s Hospital (No. 2,017,128 H) and performed in accordance with the Declaration of Helsinki.

### Study procedure

Baseline data of demographics, vital signs at admission, reasons for admission, comorbidities, laboratory, angiographic findings, and medical treatment at discharge were extracted from the medical records of Guangdong Provincial People’s Hospital. Non-fasting venous blood was drawn to evaluate NT-proBNP using an electrochemiluminescence automatic immunoassay system (Roche Eleesys ~ 2010disk, Switzerland) in the clinical laboratory. Other laboratory tests were completed in the clinical laboratory of Guangdong Provincial People’s Hospital. DM was defined as currently using anti-hypertensive treatment, being diagnosed by a physician, fasting blood glucose (FBG) ≥ 7.0mmol/L, or glycated hemoglobin A1c (HbA1C) ≥ 6.5%[14].

### Follow-up and study endpoint

Follow-ups were completed by two treated investigators blinded to the baseline data using phone-call interviews. All-cause mortality was defined as the only study endpoint because the death reasons cannot be accurately assessed through telephone visits. The length of follow-up was calculated as the death date or the last follow-up date minus the discharge date, whichever came first. All participants were followed through on June 30th, 2022.

### Statistical analysis

Subjects were first categorized as with DM and without DM according to the above criteria. Continuous variables were presented as mean value ± standard deviation (SD) or median (interquartile range) and categorical variables were presented as numbers (percentage). Differences between patients with and without DM were compared using Student’s t-test, Wilcoxon rank sum test, and chi-square test accordingly.

The Kaplan–Meier (KM) method was used to estimate the cumulative rate of all-cause mortality stratified by categorical NT-proBNP groups (≥ 1800 pg/ml and < 1800 pg/ml) and DM status (with and without DM). The Cox proportional-hazards models were conducted to calculate the hazard ratios (HRs) and 95% confidence intervals (CIs) with adjustment for covariates presented in Table 1. 5 models were constructed. Model 1 adjusted for age and sex; Model 2 adjusted for model 1 plus smoking status, heart rate, NYHA class, and reasons for admission; Model 3 adjusted for model 2 plus laboratory at admission, left ventricular ejection fraction, coronary angiography, and in-hospital percutaneous coronary intervention; Model 4 adjusted for model 3 plus comorbidities; Model 5 adjusted for model 4 plus medications at discharge. The product of DM status and NT-proBNP were included in each model to assess the interaction.

**Table 1:** Baseline Characteristics Comparison between Patients With and Without Diabetes Mellitus

Variables	Overall (N = 2287)	Without diabetes mellitus (N = 1115)	With diabetes mellitus (N = 1172)	P-value
Age (years)	63.7 ± 11.0	63.6 ± 11.6	63.7 ± 11.5	0.784
Male, n(%)	1929 (84.4)	948 (85.0)	981 (83.7)	0.385
Vital sign at admission				
Systolic blood pressure (mmHg)	125.7 ± 20.7	124.6 ± 20.2	126.8 ± 21.1	0.014
Diastolic blood pressure (mmHg)	74.4 ± 12.6	74.2 ± 12.5	74.6 ± 12.7	0.392
Heart rate (beat per minute)	79.2 ± 15.1	77.6 ± 14.7	80.7 ± 15.3	< 0.001
NYHA III-IV, n(%)	582 (25.5)	254 (22.8)	328 (28.0)	0.004
Reason for admission, n(%)				
Acute coronary syndrome	1241 (54.3)	644 (57.8)	597 (50.9)	0.001
Acute heart failure	866 (37.9)	375 (33.6)	491 (41.9)	< 0.001
Laboratory at admission				
Hemoglobin (g/L)	130.7 ± 20.5	132.3 ± 19.1	129.2 ± 21.6	< 0.001
Total cholesterol (mmol/L)	4.32 ± 1.27	4.35 ± 1.25	4.30 ± 1.28	0.372
Low-density lipoprotein cholesterol (mmol/L)	2.81 ± 0.99	2.82 ± 0.99	2.80 ± 0.99	0.481
High-density lipoprotein cholesterol (mmol/L)	0.96 ± 0.28	0.99 ± 0.30	0.93 ± 0.25	< 0.001
Triglyceride (mmol/L)	1.56 ± 1.06	1.45 ± 0.89	1.66 ± 1.18	< 0.001
Lipoprotein(a) (mg/dL)*	20.5 (10.1–43.0)	21.7 (10.4–44.2)	19.6 (9.8–40.8)	0.091
Estimated glomerular filtration rate (ml/min/1.73m^2^)*	74.6 (57.6–91.7)	76.8 (60.9–93.1)	71.9 (54.3–90.6)	< 0.001
Glycated hemoglobin A1c (%)	6.8 ± 1.7	5.7 ± 0.5	7.8 ± 1.7	< 0.001
Fasting blood glucose (mmol/L)	6.22 ± 2.60	4.91 ± 0.75	7.41 ± 3.06	< 0.001
High-sensitivity cardiac troponin-T (pg/mL)*	37.1 (18.9-183.3)	31.4 (16.5–159.0)	41.2 (20.7-204.6)	< 0.001
N-terminal B-type natriuretic peptide (pg/mL)*	1568 (619–3827)	1404 (522–3295)	1772 (724–4260)	< 0.001
Echocardiographic index				
Left ventricular ejection fraction (%)	36.3 ± 7.4	36.4 ± 7.4	36.3 ± 7.3	0.684
Coronary angiography, n(%)				
Left main	584 (25.5)	278 (24.9)	306 (26.1)	0.519
Three vessels	490 (21.4)	219 (19.6)	271 (23.1)	0.043
In-hospital percutaneous coronary intervention	1566 (68.5)	770 (69.1)	796 (67.9)	0.557
Comorbidities, n(%)				
Smoking status				< 0.001
Current	465 (20.3)	264 (23.7)	201 (17.2)	
Former	380 (16.6)	190 (17.0)	190 (16.2)	
Never	1442 (63.1)	661 (59.3)	781 (66.6)	
Hypertension	1228 (53.7)	550 (49.3)	678 (57.9)	< 0.001
Chronic kidney disease	620 (27.1)	263 (23.6)	357 (30.5)	< 0.001
Atrial fibrillation	151 (6.6)	67 (6.0)	84 (7.2)	0.265
Stroke	185 (8.1)	90 (8.1)	95 (8.1)	0.976
Myocardial infarction	884 (38.7)	432 (38.7)	452 (38.6)	0.930
Prior revascularization	1267 (55.4)	611 (54.8)	656 (56.0)	0.572
Malignant tumor	32 (1.4)	18 (1.6)	14 (1.2)	0.393
Medications at discharge, n(%)				
Dual anti-platelet	1800 (78.7)	872 (78.2)	928 (79.2)	0.569
Statins	2162 (94.5)	1065 (95.5)	1097 (93.6)	0.044
Betablocker	1908 (83.4)	924 (82.9)	984 (84.0)	0.484
Renin-angiotensin-system inhibitor	1457 (63.7)	720 (64.6)	737 (62.9)	0.401
Angiotensin receptor-neprilys inhibitor	303 (13.3)	144 (12.9)	159 (13.6)	0.646
Mineralocorticoid receptor antagonist	1101 (48.2)	508 (45.6)	593 (50.6)	0.015
Loop diuretic	1057 (46.2)	475 (42.6)	582 (49.7)	0.001
Digoxin	136 (6.0)	58 (5.2)	78 (6.7)	0.142
Calcium channel blocker	275 (12.0)	119 (10.7)	156 (13.3)	0.053
Sodium-dependent glucose transporters 2 inhibitor	119 (5.2)	2 (0.2)	117 (10.0)	< 0.001
Oral anticoagulants	530 (23.2)	240 (21.5)	290 (24.7)	0.068

* Presented as median (interquartile range)

A 1:1 ratio propensity-matched analysis with a caliper value of 0.02 was further performed to verify the robustness of the results. 29 variables, including sex, age, vital signs at admission, reasons for admission, NYHA class, coronary angiography, LVEF, laboratory, smoking status, and comorbidities, were selected based on investigator judgment for the propensity model. The propensity to DM versus non-DM was predicted by the multivariable logistic regression model, and post-estimations for propensity score-matched were evaluated by standardized bias and propensity score density [15]. Subjects were matched to the nearest available propensity score and those who have been successfully matched were removed from the model. The procedure was repeated until all DM patients were matched. 2 Cox models were then constructed. Model 1 adjusted for none, and model 2 adjusted for medications at discharge (which did not include in the propensity-matched model but had a significant impact on the mortality).

All analyses were performed using Stata version 15.1 (StataCorp LLC, College Station, TX, USA). Two-sided p-values < 0.1 for interaction tests and p-values < 0.05 for other analyses were considered statistically significant.

## Results

### Baseline characteristics of participants with and without diabetes mellitus

**Fig. 2:**
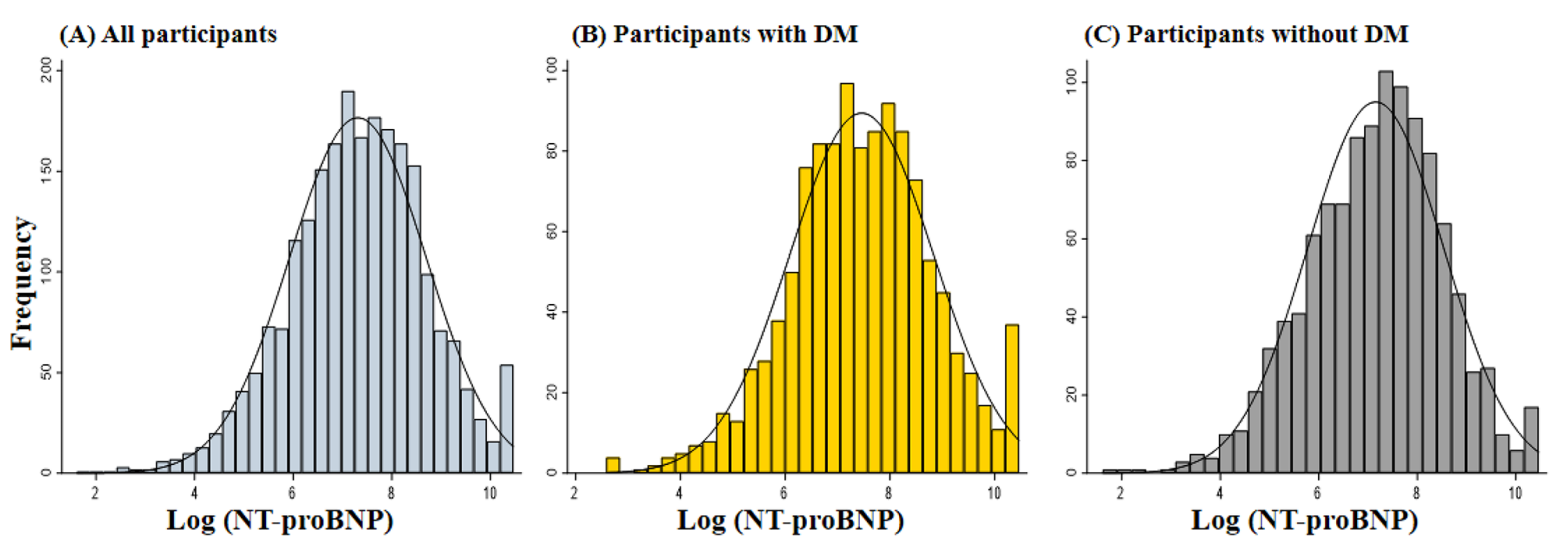
The Histograms for Log-NT-proBNP Values. (**A**) The histograms for log-NT-proBNP values in all participants. (**B**) The histograms for log-NT-proBNP values in participants with DM. (**C**) The histograms for log-NT-proBNP values in participants without DM. NT-proBNP, N-terminal pro-B-type natriuretic peptide; DM, diabetes mellitus

A total of 2287 ischemic HF patients with LVEF ≤ 50% were enrolled, and 1172 (51.2%) participants had DM. Overall, participants were on average 63.7 years old and 84.4% of them were male. The mean LVEF and median NT-proBNP were 36.3% and 1568 pg/ml, respectively. The histograms of log-NT-proBNP values for all participants (Panel A) and participants with (Panel B) and without DM (Panel C) were also displayed in Fig. 2. The most prevalent comorbidity was coronary artery disease, with 55.4% of subjects having prior revascularization, followed by hypertension (53.7%), DM (51.2%), MI (38.7%), and chronic kidney disease (CKD) (27.1%).

Compared with the DM group, patients without DM had lower heart rates, were less likely to present with acute HF and NYHA III-IV, and had higher hemoglobin and estimated glomerular filtration rate (eGFR) levels. The prevalence of triple vessel disease, hypertension, and CKD was higher in the DM group than in those without DM. Besides, patients with DM also had higher high-sensitivity cardiac troponin-T and NT-proBNP levels. (Table 1)

### NT-proBNP and all-cause mortality risk in patients with and without diabetes mellitus

**Fig. 3:**
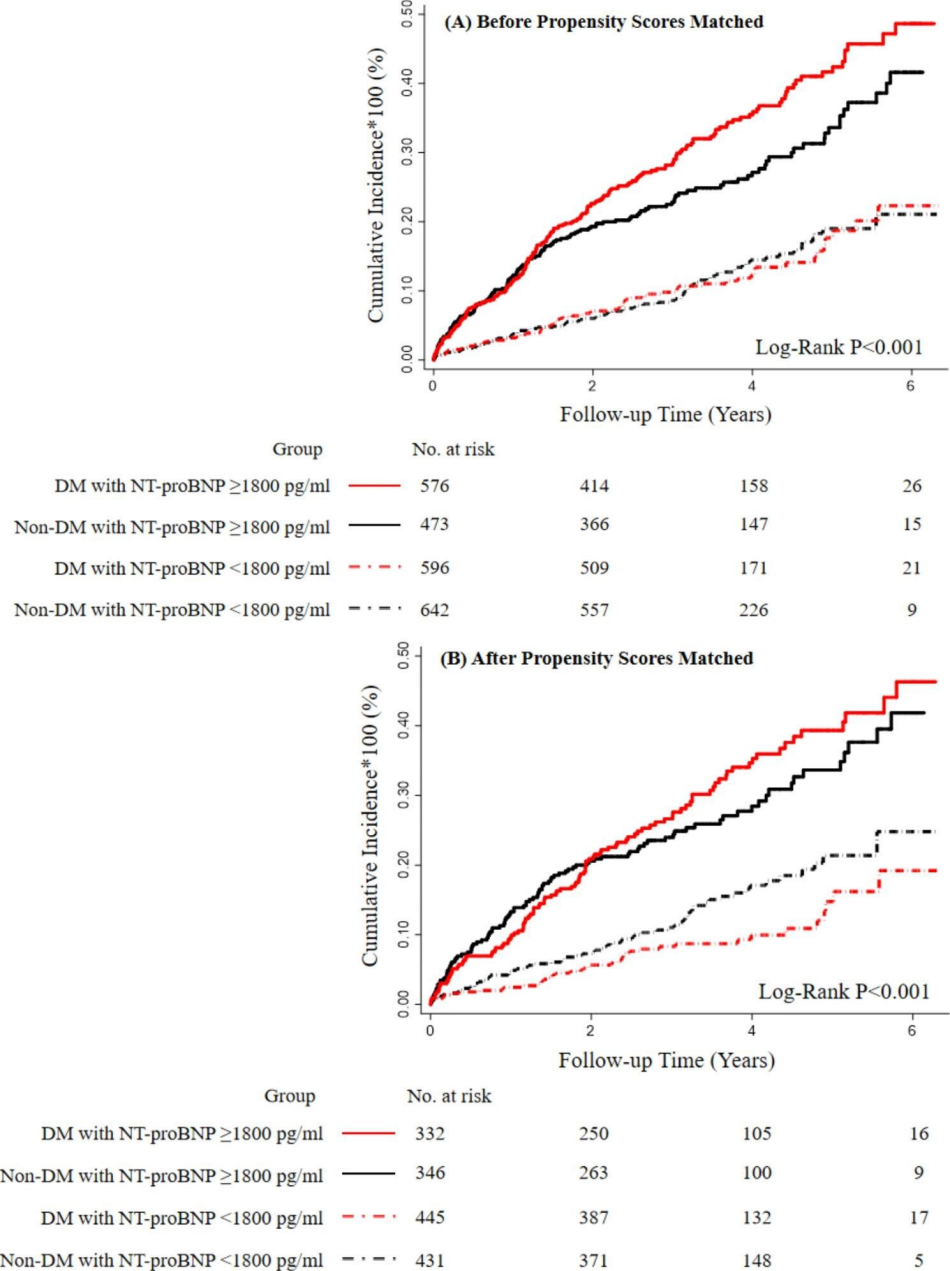
Kaplan-Meier Curve of All-cause Mortality Stratified by Categorical NT-proBNP Groups and DM status. NT-proBNP, N-terminal pro-B-type natriuretic peptide; DM, diabetes mellitus

After a median follow-up of 3.19 years (7287 person-years), 479 (20.9%) of the participants died. The incidence rates of patients with and without DM were 7.3 (6.5–8.3) and 5.8 (5.1–6.7) per 100 person-years. When NT-proBNP was considered as a continuous variable, 1-SD NT-proBNP increase was significantly associated with mortality risk in ischemic HF patients (HR: 1.46, 95% CI: 1.31–1.62) even after adjusting for the covariates, and the association was more pronounced in those with DM (HR: 1.65, 95% CI: 1.43–1.91) than those without (HR: 1.28, 95% CI: 1.09–1.50) (P-interaction = 0.016). When NT-proBNP was considered as a categorical variable, NT-proBNP ≥ 1800pg/ml was consistently and positively associated with mortality risk (HR: 1.88, 95% CI: 1.48–2.41) (Fig. 3 Panel A and Table 2). Similarly, the association was also more pronounced in those with DM (HR: 2.39, 95% CI: 1.70–3.36) than those without (HR: 1.50, 95% CI: 1.04–2.18) (P-interaction = 0.042) (Table 2).

**Table 2:** Association with NT-proBNP and All-cause Mortality in Patients with Ischemic Heart Failure According to Diabetes Mellitus Status

	Overall		Without diabetes mellitus		With diabetes mellitus		P-interaction
NT-proBNP as continuous variable (Per 1-log NT-proBNP increment)							
Event/Total (%)	479/2287 (20.9)		212/1115 (19.0)		267/1172 (22.8)		/
Incidence Rate (100 person-years)	6.6 (6.0-7.2)		5.8 (5.1–6.7)		7.3 (6.5–8.3)		/
Person-Year	7287		3645		3642		/
Model 1 (HR and 95% CI)	1.66 (1.54–1.78)		1.63 (1.36–1.69)		1.77 (1.61–1.96)		0.040
Model 2 (HR and 95% CI)	1.60 (1.48–1.73)		1.47 (1.31–1.66)		1.71 (1.54–1.90)		0.038
Model 3 (HR and 95% CI)	1.46 (1.32–1.61)		1.29 (1.11–1.50)		1.61 (1.40–1.84)		0.042
Model 4 (HR and 95% CI)	1.47 (1.33–1.63)		1.29 (1.11–1.51)		1.65 (1.44–1.90)		0.045
Model 5 (HR and 95% CI)	1.46 (1.31–1.62)		1.28 (1.09–1.50)		1.65 (1.43–1.91)		0.016
NT-proBNP as categorical variable							
NT-proBNP categorization	NT-proBNP < 1800 pg/ml	NT-proBNP ≥ 1800 pg/ml	NT-proBNP < 1800 pg/ml	NT-proBNP ≥ 1800 pg/ml	NT-proBNP < 1800 pg/ml	NT-proBNP ≥ 1800 pg/ml	
Event/Total (%)	151/1238 (12.2)	328/1049 (31.3)	80/642 (12.5)	132/473 (27.9)	71/596 (11.9)	196/576 (34.0)	/
Incidence Rate (100 person-years)	3.7 (3.1–4.3)	10.4 (9.3–11.5)	3.7 (3.0-4.6)	9.0 (7.6–10.6)	3.6 (2.9–4.6)	11.6 (10.1–13.3)	/
Person-Year	4122	3165	2172	1473	1951	1691	/
Model 1 (HR and 95% CI)	1.00 (Reference)	2.65 (2.18–3.21)	1.00 (Reference)	2.15 (1.62–2.84)	1.00 (Reference)	3.14 (2.39–4.12)	0.065
Model 2 (HR and 95% CI)	1.00 (Reference)	2.34 (1.91–2.87)	1.00 (Reference)	1.92 (1.43–2.59)	1.00 (Reference)	2.78 (2.09–3.69)	0.062
Model 3 (HR and 95% CI)	1.00 (Reference)	1.96 (1.54–2.49)	1.00 (Reference)	1.52 (1.07–2.17)	1.00 (Reference)	2.40 (1.72–3.34)	0.081
Model 4 (HR and 95% CI)	1.00 (Reference)	1.98 (1.56–2.51)	1.00 (Reference)	1.54 (1.08–2.20)	1.00 (Reference)	2.48 (1.78–3.47)	0.076
Model 5 (HR and 95% CI)	1.00 (Reference)	1.88 (1.48–2.41)	1.00 (Reference)	1.50 (1.04–2.18)	1.00 (Reference)	2.39 (1.70–3.36)	0.042

Model 1 adjusted for age and sex

Model 2 adjusted for model 1 plus smoking status, heart rate, NYHA class, and reasons for admission

Model 3 adjusted for model 2 plus laboratory at admission, left ventricular ejection fraction, coronary angiography, and in-hospital percutaneous coronary intervention

Model 4 adjusted for model 3 plus comorbidities

Model 5 adjusted for model 4 plus medications at discharge

NT-proBNP, N-terminal pro-B-type natriuretic peptide

### NT-proBNP and all-cause mortality risk in propensity-matched participants

We matched 777 patients with DM to those without DM in a 1:1 ratio. After propensity matching, difference and standardized bias in baseline characteristics between groups significantly reduce (Supplemental Figs. 1 and 2), and there was no difference in baseline characteristics between patients with and without DM (Supplemental Table 1). In the propensity matching analyses, both continuous and categorical NT-proBNP was more prominently associated with the risk of mortality in the DM group than the non-DM group. The association remained consistent even after adjusting for the prescribed medications at discharge. (Fig. 3 Panel B and Table 3)

**Table 3:** Association with NT-proBNP and All-cause Mortality in Patients with Ischemic Heart Failure according to Diabetes Mellitus Status in Propensity-matched Cohort

	Overall		Without diabetes mellitus		With diabetes mellitus		P-interaction
NT-proBNP as continuous variable (Per 1-log NT-proBNP increment)							
Event/Total (%)	315/1554 (20.3)		163/777 (21.0)		152/777 (19.6)		/
Incidence Rate (100 person-years)	6.3 (5.6-7.0)		6.5 (5.6–7.6)		6.1 (5.2–7.1)		/
Person-Year	4998		2494		2504		/
Model 1 (HR and 95% CI)	1.66 (1.51–1.81)		1.46 (1.29–1.65)		1.95 (1.69–2.24)		0.003
Model 2 (HR and 95% CI)	1.58 (1.43–1.75)		1.42 (1.25–1.63)		1.85 (1.58–2.15)		0.002
NT-proBNP as categorical variable							
NT-proBNP categorization	NT-proBNP < 1800 pg/ml	NT-proBNP ≥ 1800 pg/ml	NT-proBNP < 1800 pg/ml	NT-proBNP ≥ 1800 pg/ml	NT-proBNP < 1800 pg/ml	NT-proBNP ≥ 1800 pg/ml	
Event/Total (%)	107/876 (12.2)	208/678 (30.7)	64/431 (14.9)	99/346 (28.6)	43/445 (9.7)	109/332 (32.8)	/
Incidence Rate (100 person-years)	3.7 (3.0-4.4)	10.0 (8.7–11.5)	4.4 (3.5–5.7)	9.4 (7.7–11.4)	2.9 (2.2–3.9)	10.7 (8.8–12.9)	/
Person-Year	2922	2076	1440	1054	1482	1022	/
Model 1 (HR and 95% CI)	1.00 (Reference)	2.71 (2.14–3.42)	1.00 (Reference)	2.08 (1.52–2.85)	1.00 (Reference)	3.66 (2.57–5.21)	0.021
Model 2 (HR and 95% CI)	1.00 (Reference)	2.37 (1.85–3.03)	1.00 (Reference)	1.91 (1.36–2.67)	1.00 (Reference)	3.12 (2.15–4.53)	0.018

Model 1 adjusted for none

Model 2 adjusted for medications at discharge

NT-proBNP, N-terminal pro-B-type natriuretic peptide

## Discussion

The current study re-emphasized that higher NT-proBNP level was significantly related to all-cause mortality risk among ischemic HF patients. What’s interesting was that DM status might modify such association, indicating that NT-proBNP was more prominently associated with risk of mortality in HF patients with DM than those without. Furthermore, the results remained consistent even after adjusting for conventional risk factors or after propensity matching.

NT-proBNP is strongly associated with adverse cardiovascular outcomes and mortality in HF populations [16] and plays an important role in the diagnosis and management of HF [17, 18]. Beyond HF, NT-proBNP also has a good ability to predict death in general populations [10] and patients with DM [19]. Furthermore, a recent study from the Atherosclerosis Risk in Communities (ARIC) cohort showed that NT-proBNP discriminated mortality risk better than conventional risk factors only in subjects with DM instead of non-DM people after a median of 7.2 years of follow-up [11], which suggested that DM status may modify the relationship between NT-proBNP and risk of death. However, this study only involved general people from the western country [11], and whether the conclusions can be generalized to Chinese HF patients remains inconclusive. To the best of our knowledge, the present study was the first to investigate the relationship between NT-proBNP and mortality in ischemic HF with and without DM and demonstrated that DM status did modify this association among HF patients. The conclusions were robust even in the propensity-matching cohort.

The current study did not clarify the mechanisms underlying these observations, and the theories to explain are hypothetical. First, people with DM tend to have higher body mass index (BMI)[20] and NT-proBNP is inversely associated with BMI [21]. Hence, an equivalent increasement of NT-proBNP may reflect higher atrial pressure in DM individuals than their counterparts without DM, indirectly representing worse cardiac function among DM subjects. Second, although NT-proBNP is inversely associated with the incidence of DM [22–24], a study including 1294 individuals showed that NT-proBNP was positively associated with microvascular (HR: 1.20; 95% CI: 1.01–1.43) and macrovascular (HR: 1.37; 95% CI: 1.03–1.83) complications in those with incident DM [24], which have a deleterious impact on human health. At present, NT-proBNP and DM were deemed as partners in crime in the incidence and progression of HF [25], better clarification of the mortality risk of NT-proBNP between HF patients with different DM statuses might have a significant impact on public health and thus improve risk stratification.

DM is common in HF, and sodium-dependent glucose transporter 2 inhibitor (SGLT2i) is a promising drug and provides beneficial cardiovascular effects in DM [26] and in HF [17] in the contemporary era. The protective effects were attributed to several mechanisms, including the diuretic effect of SGLT2i, more efficient production of ATP, and mitochondrial function reestablishment [27–29]. The effect of SGLT2i on NT-proBNP was also recognized as another potential pathway to produce a beneficial impact [30, 31] but remains controversial [32]. Our current results provide robust evidence that the association between NT-proBNP and mortality was more remarkable in HF patients with DM than without, suggesting that lowering NT-proBNP levels in patients with HF and DM would be a promising direction to gain benefit. Beyond NT-proBNP, a recent study has also demonstrated that disintegrin and metalloprotease protein-17 were markedly expressed in mice with diabetic cardiomyopathy [33], which was then essential for cleaving angiotensin-converting enzyme 2 and accelerated the cardiac remodeling. Taken together, inhibition of these potential biomarkers might provide a more promising approach to the treatment of HF patients with DM.

## Limitations

The present study has some noteworthy limitations. First, this is a single-center cohort study, the study conclusions may not be generalizable to all ischemic HF patients. Second, some unmeasured and unknown confounding factors may still exist and influence the current results, although we have adjusted for multiple covariates and conducted propensity score matching analysis. Third, the current study used all-cause mortality as the only primary endpoint; therefore, whether the conclusions can be generalized to other cardiovascular outcomes is unknown. Forth, whether DM status modifies the effect of SGLT2i on NT-proBNP can not be investigated because of the low usage rate. Fifth, subjects can not be further categorized as non-DM and pre-DM due to the small sample size and inadequate power of the test.

## Conclusions

DM status modified the association between NT-proBNP and all-cause mortality in ischemic HF patients, and NT-proBNP was more prominently associated with the risk of mortality in patients with DM than those without. Future studies are needed to clarify the mechanisms underlying these observations and to investigate the drug effect on NT-proBNP in patients with and without DM.

## Electronic supplementary material

Below is the link to the electronic supplementary material.

Supplementary Material 1

Supplementary Material 2

Supplementary Material 3

## Data Availability

The deidentified participant data will be shared on a request basis. Please directly contact the corresponding author to request data sharing.

## Abbreviations

DM: diabetes mellitus
HF: heart failure
N-terminal B-type natriuretic peptide: NT-proBNP
MI: myocardial infarction
LVEF: left ventricular ejection fraction
FBG: fasting blood glucose
HbA1C: hemoglobin A1c
SD: standard deviation
HR: hazard ratio
CI: confidence interval
CKD: chronic kidney disease
eGFR: estimated glomerular filtration rate
ARIC: Atherosclerosis Risk in Communities
BMI: body mass index
SGLT2i: sodium-dependent glucose transporters 2 inhibitor
