# Supplementary material for: Diabetes mellitus status modifies the association between N-terminal B-type natriuretic peptide and all-cause mortality risk in ischemic heart failure: a prospective cohort study
Source: Diabetol Metab Syndr. 2023 Apr 11;15:72. doi: 10.1186/s13098-023-01046-5 (PMC10088130; doi:10.1186/s13098-023-01046-5)
Supplement: Supplementary file 1 — Supplementary Material 1 [file 13098_2023_1046_MOESM1_ESM.docx]

**Figure 2.** **Estimated Density of The Propensity Scores Before and After Propensity Scores Matched.**


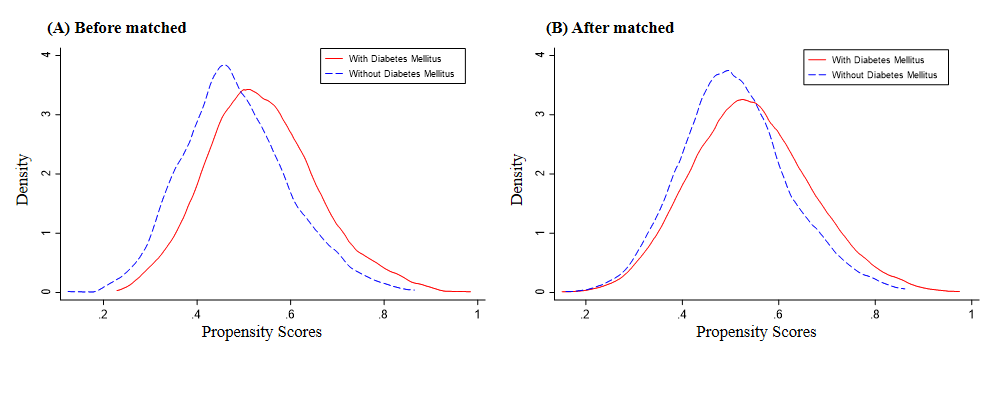


Figure 2A. Estimated Density of The Propensity Scores Before Propensity Scores Matched.

Figure 2B. Estimated Density of The Propensity Scores After Propensity Scores Matched.
