# Supplementary material for: Diabetes mellitus status modifies the association between N-terminal B-type natriuretic peptide and all-cause mortality risk in ischemic heart failure: a prospective cohort study
Source: Diabetol Metab Syndr. 2023 Apr 11;15:72. doi: 10.1186/s13098-023-01046-5 (PMC10088130; doi:10.1186/s13098-023-01046-5)
Supplement: Supplementary file 2 — Supplementary Material 2 [file 13098_2023_1046_MOESM2_ESM.docx]

**Figure 3.** **Standardized Bias across Covariates Before and After Propensity-matched.**


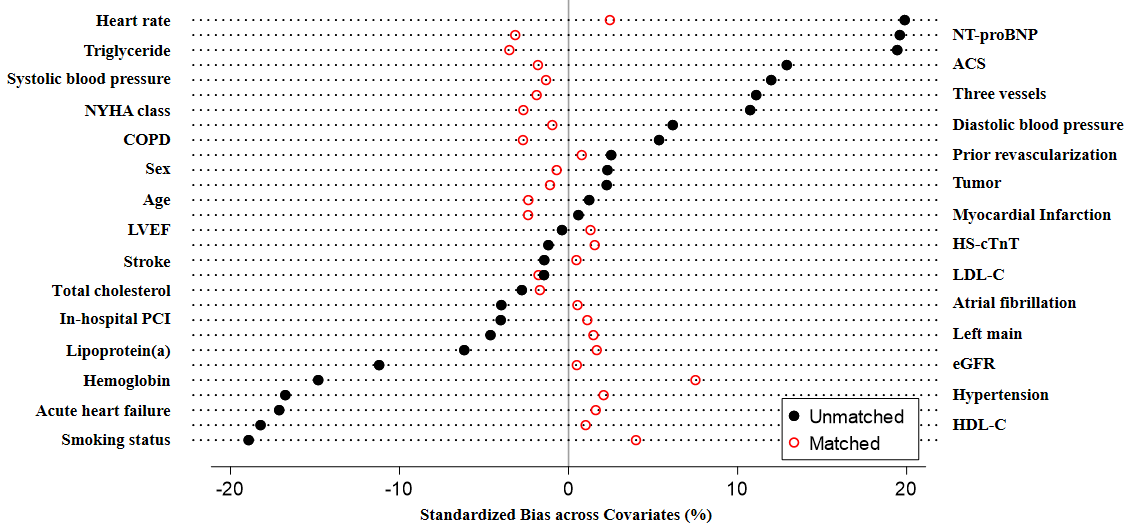


NT-proBNP, N-terminal pro-B-type natriuretic peptide; ACS, acute coronary syndrome; NYHA, New York Heart Association; COPD, chronic obstructive pulmonary disease; LVEF, left ventricular ejection fraction; HS-cTnT, high-sensitive cardiac troponin T; LDL-C, low-density lipoprotein cholesterol; PCI, percutaneous coronary intervention; eGFR, estimated glomerular filtration rate; HDL-C, high-density lipoprotein cholesterol
