# Supplementary material for: Diabetes mellitus status modifies the association between N-terminal B-type natriuretic peptide and all-cause mortality risk in ischemic heart failure: a prospective cohort study
Source: Diabetol Metab Syndr. 2023 Apr 11;15:72. doi: 10.1186/s13098-023-01046-5 (PMC10088130; doi:10.1186/s13098-023-01046-5)
Supplement: Supplementary file 3 — Supplementary Material 3 [file 13098_2023_1046_MOESM3_ESM.docx]

**Supplemental Table 1. Baseline Characteristics Comparison between Patients With and Without Diabetes Mellitus after Propensity Score Matching.**

| Variables | Overall  (N=1554) | Without diabetes mellitus (N=777) | With diabetes mellitus (N=777) | P-value |
| --- | --- | --- | --- | --- |
| Age (years) | 63.8±11.0 | 63.9±11.6 | 63.7±10.5 | 0.637 |
| Male, n(%) | 1318 (84.8) | 658 (84.7) | 660 (84.9) | 0.888 |
| Vital sign at admission |  |  |  |  |
| Systolic blood pressure (mmHg) | 125.8±20.7 | 126.0±20.7 | 125.7±20.6 | 0.792 |
| Diastolic blood pressure (mmHg) | 75.0±12.5 | 75.1±12.5 | 74.9±12.8 | 0.860 |
| Heart rate (beat per minute) | 78.8±14.8 | 78.6±14.6 | 79.0±14.9 | 0.624 |
| NYHA Ⅲ-Ⅳ, n(%) | 375 (24.1) | 192 (24.7) | 183 (23.6) | 0.594 |
| Reason for admission, n(%) |  |  |  |  |
| Acute coronary syndrome | 873 (56.2) | 433 (55.7) | 440 (56.6) | 0.720 |
| Acute heart failure | 562 (36.2) | 284 (36.5) | 278 (35.8) | 0.751 |
| Laboratory at admission |  |  |  |  |
| Hemoglobin (g/L) | 132.3±19.2 | 131.6±18.9 | 133.1±19.5 | 0.122 |
| Total cholesterol (mmol/L) | 4.33±1.25 | 4.34±1.24 | 4.32±1.26 | 0.737 |
| Low-density lipoprotein cholesterol (mmol/L) | 2.83±0.98 | 2.84±0.97 | 2.82±0.99 | 0.726 |
| High-density lipoprotein cholesterol (mmol/L) | 0.95±0.25 | 0.95±0.23 | 0.96±0.26 | 0.823 |
| Triglyceride (mmol/L) | 1.51±0.88 | 1.53±0.98 | 1.49±0.77 | 0.406 |
| Lipoprotein(a) (mg/dL)* | 20.1 (9.9-41.0) | 20.8 (10.2-40.6) | 19.3 (9.7-41.2) | 0.620 |
| Estimated glomerular filtration rate (ml/min/1.73m^2^)* | 75.1 (59.2-91.5) | 74.7 (58.9-88.9) | 75.7 (59.3-94.0) | 0.085 |
| Glycated hemoglobin A1c (%) | 6.8±1.6 | 5.7±0.5 | 7.8±1.7 | <0.001 |
| Fasting blood glucose (mmol/L) | 6.04±2.31 | 4.91±0.75 | 7.14±2.74 | <0.001 |
| High-sensitivity cardiac troponin-T (pg/mL)* | 35.6 (18.4-160.1) | 32.9 (17.7-157.1) | 36.8 (18.9-172.1) | 0.159 |
| N-terminal B-type natriuretic peptide (pg/mL)* | 1468 (597-3345) | 1496 (580-3406) | 1397 (625-3312) | 0.982 |
| Echocardiographic index |  |  |  |  |
| Left ventricular ejection fraction (%) | 36.2±7.4 | 36.2±7.5 | 36.3±7.3 | 0.800 |
| Coronary angiography, n(%) |  |  |  |  |
| Left main | 403 (25.9) | 204 (26.3) | 199 (25.6) | 0.772 |
| Three vessels | 322 (20.7) | 164 (21.1) | 158 (20.3) | 0.707 |
| In-hospital percutaneous coronary intervention | 1064 (68.5) | 530 (68.2) | 534 (68.7) | 0.827 |
| Comorbidities, n(%) |  |  |  |  |
| Smoking status |  |  |  | 0.632 |
| Current | 315 (20.3) | 150 (19.3) | 165 (21.2) |  |
| Former | 247 (15.9) | 126 (16.2) | 121 (15.6) |  |
| Never | 992 (63.8) | 501 (64.5) | 491 (63.2) |  |
| Hypertension | 832 (53.5) | 420 (54.1) | 412 (53.0) | 0.684 |
| Chronic kidney disease | 405 (26.1) | 204 (26.3) | 201 (25.9) | 0.862 |
| Atrial fibrillation | 95 (6.1) | 48 (6.2) | 47 (6.1) | 0.916 |
| Stroke | 127 (8.2) | 63 (8.1) | 64 (8.2) | 0.926 |
| Myocardial infarction | 589 (37.9) | 290 (37.3) | 299 (38.5) | 0.638 |
| Prior revascularization | 863 (55.5) | 430 (55.3) | 433 (55.7) | 0.878 |
| Malignant tumor | 23 (1.5) | 11 (1.4) | 12 (1.5) | 0.834 |
| Medications at discharge, n(%) |  |  |  |  |
| Dual anti-platelet | 1224 (78.8) | 605 (77.9) | 619 (79.7) | 0.385 |
| Statins | 1482 (95.4) | 745 (95.9) | 737 (94.9) | 0.334 |
| Betablocker | 1310 (84.3) | 651 (83.8) | 659 (84.8) | 0.577 |
| Renin-angiotensin-system inhibitor | 992 (63.8) | 504 (64.9) | 488 (62.8) | 0.398 |
| Angiotensin receptor-neprilys inhibitor | 198 (12.7) | 102 (13.1) | 96 (12.4) | 0.648 |
| Mineralocorticoid receptor antagonist | 748 (48.1) | 375 (48.3) | 373 (48.0) | 0.919 |
| Loop diuretic | 708 (45.6) | 351 (45.2) | 357 (46.0) | 0.760 |
| Digoxin | 93 (6.0) | 50 (6.4) | 43 (5.5) | 0.454 |
| Calcium channel blocker | 178 (11.5) | 89 (11.5) | 89 (11.5) | 1.000 |
| Sodium-dependent glucose transporters 2 inhibitor | 76 (4.9) | 2 (0.2) | 74 (9.5) | <0.001 |
| Oral anticoagulants | 349 (22.5) | 171 (22.0) | 178 (22.9) | 0.670 |

* Presented as median (interquartile range).
